# Sixteen-Year Interval CT Comparison in Chiari II Malformation After Early Ventriculoperitoneal Shunting: Marked Ventricular Improvement and a Prolonged Seizure-Free Interval

**DOI:** 10.7759/cureus.102802

**Published:** 2026-02-01

**Authors:** Ahmed H Khird, Alaa A Alotaibi

**Affiliations:** 1 Neurology, King Abdulaziz University Faculty of Medicine, Jeddah, SAU

**Keywords:** agenesis of the corpus callosum, chiari ii malformation, epilepsy, hydrocephalus, myelomeningocele, schizencephaly, seizure recurrence, ventricular remodeling, ventriculomegaly, ventriculoperitoneal shunt

## Abstract

Chiari II malformation is commonly associated with hydrocephalus requiring early cerebrospinal fluid (CSF) diversion and may coexist with complex supratentorial developmental abnormalities. As a result, patients with severe congenital structural brain anomalies and long-term ventriculoperitoneal (VP) shunting often experience neurodevelopmental impairment and are at increased risk of epilepsy. We report a 17-year-old girl with Chiari II malformation, repaired myelomeningocele, and ventriculoperitoneal shunting since birth (revision at five years of age), demonstrating marked ventricular improvement over a 16-year interval and a prolonged seizure-free interval despite severe congenital supratentorial malformations.

## Introduction

Chiari II malformation is a congenital hindbrain disorder characterized by the downward herniation of the cerebellum and brainstem through the foramen magnum, most commonly in association with open spina bifida (myelomeningocele), with characteristic MRI findings involving the posterior fossa and supratentorial intracranial structures [1]. It is frequently complicated by hydrocephalus requiring early cerebrospinal fluid (CSF) diversion [2]. Neurological morbidity in this population may include epilepsy, which has been reported in myelomeningocele cohorts [3]. Epilepsy is also well recognized in children with shunt-treated hydrocephalus; in one large cohort, approximately one-third (32%) developed epilepsy [4]. Beyond posterior fossa abnormalities, supratentorial developmental anomalies are also common; fetal MRI studies in myelomeningocele have demonstrated a high prevalence of supratentorial anomalies [5]. Ventriculoperitoneal (VP) shunting reduces ventricular dilation by diverting cerebrospinal fluid, and long-term diversion may be associated with gradual ventricular remodeling and variable long-term outcomes [6,7].

Given these typical expectations, prolonged seizure freedom, especially after the discontinuation of antiseizure medication, in a patient with severe congenital supratentorial malformations would be considered uncommon and clinically notable. In this report, we compare two brain computed tomography (CT) studies performed 16 years apart following early VP shunting and describe marked interval improvement in supratentorial ventriculomegaly together with a prolonged seizure-free interval, highlighting that favorable long-term structural change and seizure control may occur despite extensive congenital neuroanatomical abnormalities [8].

## Case presentation

A 17-year-old girl with Chiari II malformation and hydrocephalus presented to the emergency department after experiencing multiple generalized seizures over several hours following a prolonged seizure-free interval. She had undergone ventriculoperitoneal (VP) shunt insertion at birth for hydrocephalus associated with repaired myelomeningocele, with shunt revision at five years of age. Her past medical history was significant for chronic bilateral lower-limb weakness with wheelchair dependence. Additional comorbidities included neurogenic bladder with urinary incontinence and learning difficulties. At baseline, she was conscious and interactive (Glasgow Coma Scale {GCS}: 15/15), mobilized by crawling or wheelchair, and was independent in feeding, dressing, and bathing.

She underwent VP shunt revision at five years of age (reported as an emergency procedure for suspected shunt malfunction), after which she developed recurrent seizures described as repetitive eye blinking/twitching, followed by generalized stiffening and jerky movements of the extremities lasting less than one minute. Neuroimaging from the time of shunt revision was not available for inclusion due to archival limitations. She was treated with carbamazepine syrup with improvement in seizure frequency, and the medication was gradually tapered at seven years of age due to sustained seizure control. She then remained seizure-free for many years. The only reported breakthrough seizure occurred approximately two years prior to the current presentation while eating; it was self-limited, and the family did not seek medical evaluation at that time. No further episodes were reported until the current illness.

During the current episode, the patient developed recurrent seizures characterized by head deviation to the left and repetitive eye blinking with oroalimentary automatisms, followed by generalized stiffening and the rhythmic jerking of all four limbs associated with urinary incontinence. Each event lasted approximately 1-2 minutes, resolved spontaneously, and was followed by postictal fatigue. Multiple similar seizures occurred within a short period, prompting emergency department evaluation.

On arrival, she was alert and oriented (GCS: 15/15). Cranial nerve examination was unremarkable, with equal reactive pupils, intact extraocular movements without nystagmus or diplopia, and no facial asymmetry. Motor examination demonstrated normal tone and full strength in the upper limbs (5/5) without drift. The lower limbs showed chronic spasticity (left greater than right) with a flexed posture; she was able to move both lower limbs against gravity, consistent with her baseline neurological status. Sensation was intact to pinprick in all extremities, and reflexes were preserved. Routine blood investigations did not reveal a major metabolic derangement that would independently explain seizure clustering.

**Figure 1:**
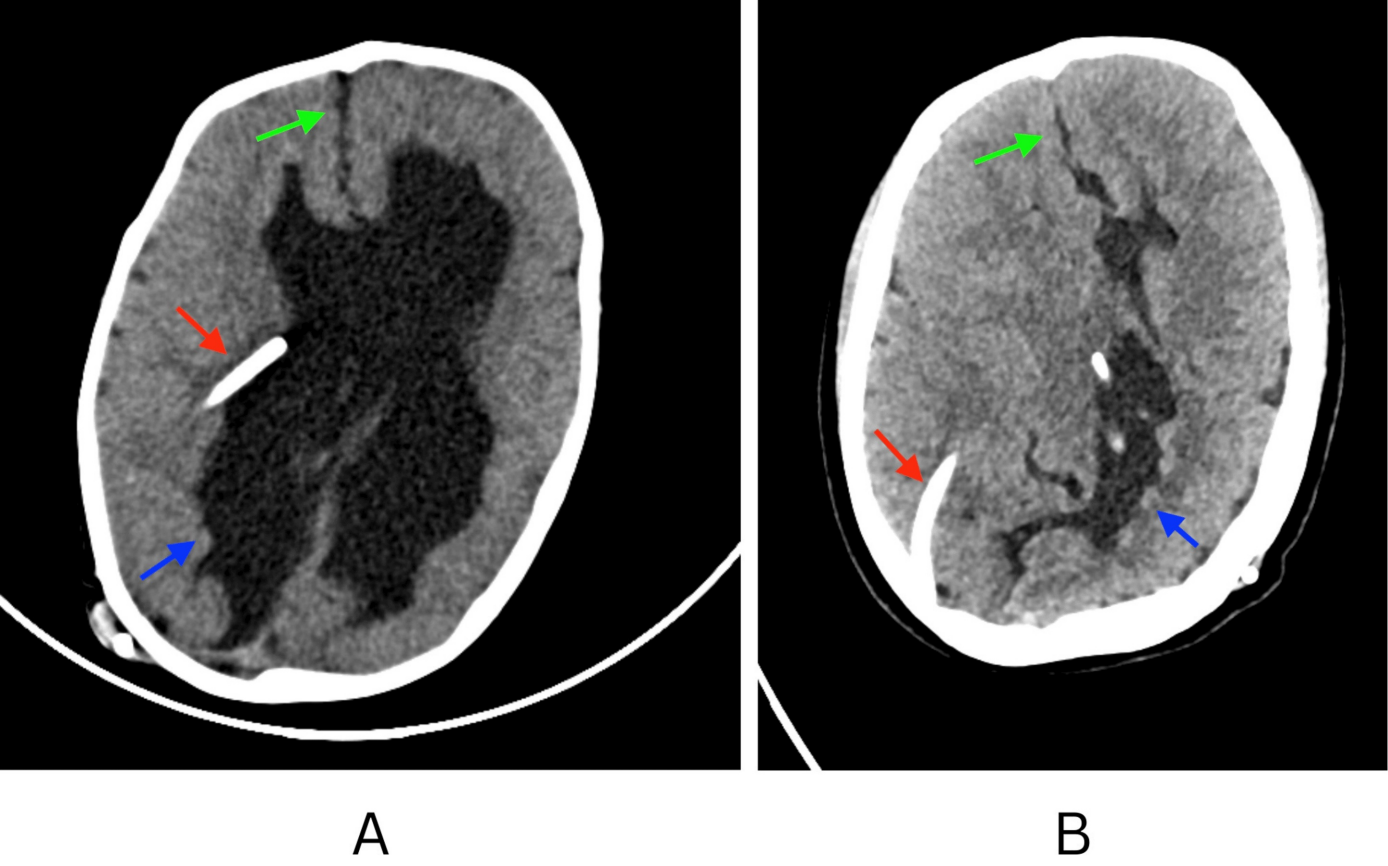
Non-contrast CT of the brain comparison between 2009 (A) and 2025 (B) demonstrating marked interval improvement in supratentorial ventriculomegaly over 16 years. Red arrows indicate the VP shunt catheter. Green arrows highlight midline findings consistent with corpus callosum agenesis with gyral interdigitation. Blue arrows indicate a right cerebral CSF cleft consistent with schizencephaly, persisting despite ventricular remodeling. CT, computed tomography; VP, ventriculoperitoneal; CSF, cerebrospinal fluid

Given the patient’s history of lifelong VP shunting and the need to assess ventricular size and underlying neuroanatomy, a non-contrast computed tomography (CT) of the brain was performed (October 2025). CT demonstrated a VP shunt catheter in situ with no acute territorial infarction, intracranial hemorrhage, mass effect, herniation, or hydrocephalus. Importantly, direct comparison with the oldest available CT from 2009 demonstrated marked interval improvement in supratentorial ventriculomegaly over a 16-year period (Figure 1). Despite the reduction in ventricular caliber, severe congenital supratentorial abnormalities persisted, predominantly involving the right cerebral hemisphere, including a right cerebral CSF cleft lined by gray matter consistent with closed-lip schizencephaly and the complete agenesis of the corpus callosum with gyral interdigitation. These congenital anomalies are best appreciated on MRI; however, MRI images were not available for inclusion in this report. MRI is recommended when feasible for the optimal characterization of Chiari II-associated supratentorial malformations.

The patient received an intravenous levetiracetam loading dose (1.5 g) for seizure clustering and was admitted for observation and neurological monitoring. Neurosurgical consultation was obtained, and maintenance levetiracetam 500 mg orally twice daily was initiated. Following levetiracetam loading, no further clinical seizures were observed during admission, and she remained at her neurological baseline. She was discharged on levetiracetam 500 mg twice daily with seizure precautions and outpatient neurology follow-up and was advised to return urgently for recurrent seizures, altered mental status, or new/worsening neurological symptoms.

## Discussion

The long-term course of Chiari II malformation depends on multiple factors, including the severity of hydrocephalus, the presence of associated supratentorial developmental abnormalities, and the effectiveness of cerebrospinal fluid diversion [8]. Supratentorial anomalies are frequently present in Chiari II malformation and open spina bifida and may contribute to long-term neurological morbidity [1,5]. Early VP shunting plays a central role in controlling hydrocephalus and may be associated with gradual ventricular remodeling and variable long-term outcomes [6,7]. However, patients with complex congenital structural brain anomalies often experience neurodevelopmental impairment and remain at an increased risk of epilepsy and recurrent seizures, particularly among individuals with myelomeningocele and associated hydrocephalus requiring shunt diversion [2,3]. Epilepsy has also been described in children with shunted hydrocephalus, reinforcing the recognized seizure risk in this population [4].

We have presented a case of a 17-year-old girl with Chiari II malformation and VP shunting since the first day of life who demonstrated marked interval improvement in supratentorial ventriculomegaly over a 16-year imaging interval compared to childhood CT, despite persistent severe supratentorial malformations. The patient also exhibited a prolonged seizure-free interval for many years following childhood seizures and medication discontinuation, highlighting a more favorable long-term seizure course than typically anticipated in patients with extensive congenital brain abnormalities [3,4]. This case reinforces the importance of direct comparison to historical neuroimaging in patients with complex congenital neuroanatomy to accurately assess long-term structural change and clinical trajectory.

## Conclusions

This case demonstrates that a substantial long-term reduction in ventriculomegaly can occur in Chiari II malformation following early ventriculoperitoneal shunting, even in the presence of severe associated supratentorial malformations. Clinically, the prolonged seizure-free interval observed suggests that seizure prognosis in this population may be more variable than commonly assumed, and long-term seizure freedom can occur despite extensive congenital neuroanatomical abnormalities. For surgeons and clinicians managing Arnold-Chiari type II, this highlights the value of timely cerebrospinal fluid diversion, sustained surveillance for shunt function, and the careful longitudinal interpretation of ventricular size in the context of each patient’s baseline anatomy. In patients presenting later with seizure recurrence or acute neurological symptoms, comparison to early baseline imaging can assist in distinguishing true hydrocephalus progression or shunt malfunction from stable congenital findings, thereby guiding appropriate neurosurgical decision-making and follow-up. Future studies incorporating systematic long-term imaging and standardized seizure outcome reporting in shunted Chiari II cohorts may help identify predictors of favorable ventricular remodeling and seizure trajectories and refine counseling and management strategies.
